# Hierarchical decision‐making balances current and future reproductive success

**DOI:** 10.1111/mec.14583

**Published:** 2018-04-23

**Authors:** Eva Ringler, Georgine Szipl, Ryan J. Harrigan, Perta Bartl‐Binder, Rosanna Mangione, Max Ringler

**Affiliations:** ^1^ Department of Integrative Biology and Physiology University of California Los Angeles Los Angeles California; ^2^ Messerli Research Institute University of Veterinary Medicine Vienna, Medical University of Vienna, University of Vienna Vienna Austria; ^3^ Department of Integrative Zoology University of Vienna Vienna Austria; ^4^ Core Facility KLF for Behaviour and Cognition University of Vienna Vienna Austria; ^5^ Center for Tropical Research Institute of the Environment and Sustainability University of California Los Angeles California; ^6^ Haus des Meeres Aqua Terra Zoo GmbH Vienna Austria; ^7^ Department of Ecology and Evolutionary Biology University of California Los Angeles Los Angeles California

**Keywords:** *Allobates femoralis*, behavioural flexibility, parental care, resource use, tadpole transport

## Abstract

Parental decisions in animals are often context‐dependent and shaped by fitness trade‐offs between parents and offspring. For example, the selection of breeding habitats can considerably impact the fitness of both offspring and parents, and therefore, parents should carefully weigh the costs and benefits of available options for their current and future reproductive success. Here, we show that resource‐use preferences are shaped by a trade‐off between parental effort and offspring safety in a tadpole‐transporting frog. In a large‐scale in situ experiment, we investigated decision strategies across an entire population of poison frogs that distribute their tadpoles across multiple water bodies. Pool use followed a dynamic and sequential selection process, and transportation became more efficient over time. Our results point to a complex suite of environmental variables that are considered during offspring deposition, which necessitates a highly dynamic and flexible decision‐making process in tadpole‐transporting frogs.

## 1 INTRODUCTION

How animals use resources is constrained by the distribution of resources in space and time (Bell, 1991). Based on previous experience and currently available information, individuals need to be flexible when deciding whether to exploit known resources or to explore their surroundings for new ones (Cohen, McClure, & Yu, 2007). The costs of the acquisition and exploitation of a specific resource, in terms of energy expenditure and time spent, will increase with decreasing predictability and availability, and with increasing demand and competition, and these costs drive the evolution of optimal resource‐use strategies (Bell, 1991). Generally, it is assumed that stable and predictable environments favour the development of stereotypic behaviour and rather low levels of flexibility, while the ability to dynamically adapt individual preferences and decision rules is assumed to be one of the most important requirements when living in complex and unpredictable environments (Piersma & van Gils, 2011). Individual and consensus decision‐making have been studied in a variety of taxa (McFarland, 1977), with a strong focus on mating (Rosenthal, 2017) and foraging strategies (O'Brien, Browman, & Evans, 1990), but decision‐making during parental care has previously received less attention (but see Neff, 2003; Ringler, Pašukonis, et al., 2015; Ringler, Hödl, & Ringler, 2015; Zöttl, Chapuis, Freiburghaus, & Taborsky, 2013).

In the context of reproduction, many animals require specific display sites for courtship, suitable oviposition sites to deposit their eggs or nest sites where they care for their offspring. The availability of these nontrophic reproductive resources can considerably limit individual fitness and population density (Alonso‐Alvarez & Velado, 2012) and may thus drive the evolution of optimal resource‐use strategies. Empirical and theoretical work had shown that parental care not only provides benefits to current offspring, but can result in considerable costs to the parent in terms of energy expenditure, survival likelihood and reduced future reproductive success in iteroparous species. As a consequence, parents are expected to optimize their decisions concerning parental effort and reproductive resource use when facing this trade‐off (Kilner & Hinde, 2012; Trivers, 1972). While some reproductive resources may be found within the habitat of the adult individuals, many species use specific habitats or resources exclusively for breeding and are required to conduct repeated and extended movements between those locations.

In amphibians, due to their complex life cycle, with eggs and tadpoles that strongly depend on water, and a mostly terrestrial adult stage (Wells, 2007), most species require very specific reproductive resources/habitats that are often distinct from their adult habitats. Many amphibians from temperate regions show strong natal breeding philopatry with annual movements between terrestrial and aquatic sites for breeding (for a review see Sinsch, 2014). They often return to natal sites, even when a closer suitable breeding habitat is available (Husté, Clobert, & Miaud, 2006; Joly & Miaud, 1989) or even long after the aquatic sites had been destroyed (Heusser, 1960), suggesting a stereotypic and probably innate preference for breeding sites (but see also Petranka & Holbrook, 2006). This is probably the result of strong selective pressure on the reproductive timing, exerted by the strict and predictable seasonal climatic conditions (Rowe & Ludwig, 1991) and comparatively low variation and fluctuation in available breeding sites. Thus, temperate amphibians benefit from selecting the most predictable water body, which in many cases will be the natal pond or stream (Hartel, Sas, Pernetta, & Geltsch, 2007). In contrast, many tropical frogs depend on small and ephemeral water bodies for reproduction (Duellman, 1989), and high structural complexity and rapid local fluctuations in tropical habitats probably decrease the adaptive value to attempt re‐using natal water bodies. To date, there is very little information available on strategies for resource exploitation and the decision rules underlying these movement patterns in tropical amphibians, particularly in natural settings (but see Beck, Loretto, Ringler, Hödl, & Pašukonis, 2017; von May, Medina‐Müller, Donnelly, & Summers, 2009; Schulte et al., 2011).

Poison frogs (Dendrobatidae, Aromobatinae, Santos et al., 2009) are common inhabitants of Neotropical rainforests that may employ dynamic decision‐making due to unpredictable resource availability. Their life history is characterized by a prolonged breeding period, diurnal activity, a high degree of territoriality and site fidelity, prolonged courtship, terrestrial oviposition and obligatory tadpole transport to aquatic sites (Pröhl, 2005; Wells, 2007; Weygoldt, 1987). The sites used for tadpole deposition range from very small water bodies (e.g., inside bromeliads) to moderate‐sized puddles and large floodplains (Brown, Morales, & Summers, 2010; Brown, Twomey, Morales, & Summers, 2008). The trade‐off between persistence, food availability and predator presence, all mediated by pool size, likely was a key factor in the evolution of diverse mating systems among the poison frogs (Brown et al., 2008, 2010). Many experimental studies have shown that frogs are able to assess the quality of water bodies in terms of predation risk and competition among offspring (McKeon & Summers, 2013; Rojas, 2014; Schulte et al., 2011), but our knowledge of the behavioural strategies involved in site selection is still limited (Buxton & Sperry, 2016).

To better understand the resource‐use patterns and underlying decision‐making strategies used by animals that live in unpredictable environments, we conducted a large‐scale, controlled, in situ experiment on a tropical poison frog. Our study species, the dendrobatid frog *Allobates femoralis,* is widely distributed across the Amazon basin and Guyana shield. During the reproductive period that coincides with local rainy seasons (Kaefer, Montanarin, da Costa, & Lima Pimentel, 2012; French Guiana: December to July, Bongers, Charles‐Dominique, Forget, & Théry, 2001), males call from elevated structures on the forest floor to announce territory possession to male competitors and to attract females (Roithmair, 1992). Pair formation, courtship and mating take place in the male's territory (Ringler, Ringler, Jehle, & Hödl, 2012; Roithmair, 1992), where externally fertilized terrestrial clutches of approximately 20 eggs are laid in the leaf litter. Both sexes are highly iteroparous; females can deposit one clutch on average every 8 days (Weygoldt, 1980), and males have been observed to attend to up to five clutches simultaneously (Ursprung, Ringler, Jehle, & Hödl, 2011). After 3–4 weeks of larval development, males transport hatched tadpoles to aquatic sites up to 200 m away (Ringler, Pašukonis, Hödl, & Ringler, 2013b) and usually return to their territories, where they might have further clutches (Beck et al., 2017; Ringler et al., 2012). Tadpoles are deposited in a variety of medium‐sized terrestrial water bodies, such as floodplains, water‐filled depressions, palm fronds and holes in fallen trees. Males have been observed to distribute tadpoles across several water bodies, which likely acts as a bet‐hedging strategy against total offspring loss (Erich, Ringler, Hödl, & Ringler, 2015), and do not provide any further care after tadpole deposition. Recent studies have shown that suitable water sites for tadpole deposition seem to be a limiting resource in *A. femoralis* (Ringler, Hödl, et al., 2015) and that spatial memory plays an important role in the reproductive behaviour of this species (Beck et al., 2017; Pašukonis, Warrington, Ringler, & Hödl, 2014; Pašukonis et al., 2016). Transporting males usually follow straight trajectories to and between deposition sites (Beck et al., 2017; Pašukonis et al., 2017). This is indicative of active decision‐making prior to the actual transporting movement rather than a random or guided search to find deposition sites. After metamorphosis is completed, emerging froglets disperse into the habitat, resulting in adult individuals ending up at a wide range of distances from their natal water pools (M. Ringler, unpublished data, see also Figure S5 in the supplementary materials). As reproductive maturity is reached in the following breeding season, and annual survival of adults is quite low (Ursprung et al., 2011), typically we find consecutive cohorts of parents and their respective offspring in subsequent years with relatively little overlap of adult generations.

Given the alleged costs of tadpole transport, in terms of energy expenditure and time investment, we expect that efficient exploitation and search strategies have evolved for tadpole transport in *A. femoralis*. Theory suggests that organisms preferentially use habitats and resources that maximize their individual fitness (Fretwell & Lucas, 1969), that is minimize the risk to their own and their offspring's survival. As *A. femoralis* does not provide any further parental care such as feeding after tadpole deposition, pool selection likely represents a pivotal decision severely impacting an offspring's chances for survival.

We aimed to test the following (not mutually exclusive) resource‐use scenarios:

1. *Natal breeding philopatry*: Individuals have a strong preference for their natal pool, particularly early in the breeding season, when not much additional information about alternative options has yet been acquired.
2. *Close to home*: If individuals are trying to maximize future reproductive success by minimizing parental efforts, they should preferentially choose breeding sites near their current location. This of course requires a certain knowledge of the surrounding habitat, which has been recently demonstrated for *A. femoralis* (Pašukonis et al., 2014, 2016).
3. *Predator avoidance*: If individuals try to maximize current reproductive success, they should avoid sites that contain predators of their offspring.

Resource selection might be performed in any one of these scenarios, or it might follow a flexible and stepwise decision process, in which individuals first approach preferred sites but will only make the final decision on the spot, depending on the prevailing conditions. Moreover, if individuals update their information about available resources and adapt their future deposition strategies accordingly, we should see an optimization of transporting performance (i.e., reduced energy and time expenditure) over time.

**Figure 1:**
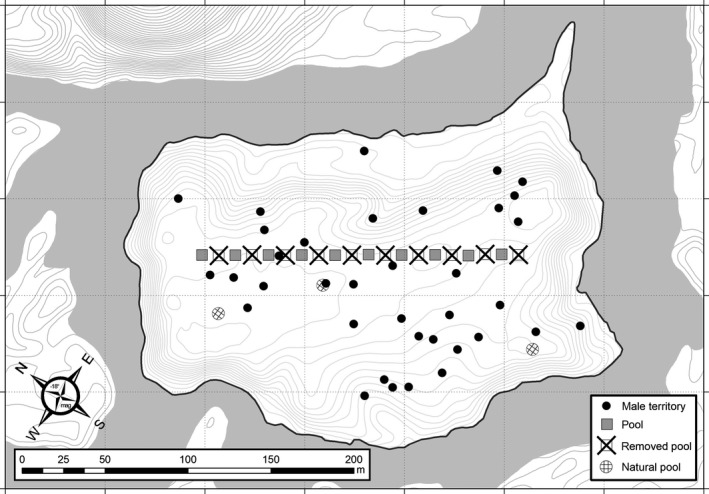
Schematic island set‐up. Every second artificial pool (crossed squares) was removed in fall 2012 after all founder tadpoles had emerged, leaving 10 pools (solid squares) at the onset of the breeding season of 2013. Circles with squared grids indicate locations of forest pools. Dots represent the distribution of male territories (centroids of individual capture points) across the island as recorded during the first sampling event in January 2013

To investigate whether tadpole deposition strategies in *A. femoralis* follow such a flexible process, we designed a large‐scale field experiment using an introduced poison frog population confined to a river island (Figure 1), artificial water bodies, molecular parentage assignments, GIS analyses, as well as decision tree and random forest modelling. Using this integrative approach, we were able to identify a large number of tadpole deposition events from all successfully breeding males in the population and to assess the influence of factors such as predator presence and spatial distances. By conducting a controlled deposition study across multiple generations, we hope to better understand how risks and benefits are processed and assessed, decisions are adjusted, and reproductive success is maximized in tropical tadpole‐transporting amphibians.

## 2 MATERIALS AND METHODS

### 2.1 Ethics and permits

This study was approved by the scientific committee of the “Nouragues Ecological Research Station.” All necessary authorizations for tissue sampling were provided by the “Centre National de la Recherche Scientifique Guyane” (CNRS Guyane, permit numbers: 12/05/2009 and 16/12/2009) and by the “Direction Régionale de l'Environment de Guyane” (DIREN, permit number: arrêté no./2010–015). All sampling was conducted in strict accordance with current French and EU law and followed the ASAB guidelines.

### 2.2 Study population

The study was conducted in an experimental *A. femoralis* population that had been introduced in a controlled manner on a 5‐ha river island (Ringler et al., 2016) in a lowland rainforest near “Saut Pararé” field camp (4°02′N, 52°41′W) of the CNRS “Nouragues Ecological Research Station” in the nature reserve “Les Nouragues,” French Guiana. In March 2012, we collected 1,800 *A. femoralis* tadpoles from artificial pools on the adjacent mainland (Ringler, Pašukonis, et al., 2015; Ringler, Hödl, et al., 2015), took a small tail clip as a tissue sample and released them on the river island, which was previously uninhabited by this species (Ringler, Mangione, & Ringler, 2014). We distributed the tadpoles across 20 artificial plastic pools (volume ~15 L, interpool distance 10 m, 90 tadpoles per pool, Figure 1) that were dug level into the ground and filled by rain, and recorded this “natal pool” for every tadpole. In fall 2012, after all tadpoles had emerged, every second pool was removed.

### 2.3 Sampling

From January to March 2013, we conducted extensive daily surveys on the island between ~08:00 and 19:00 hr. By continuously sampling all individuals encountered during the daily surveys, we attempted total sampling of all males and females on the island, and we assessed the sampling coverage by calculating cumulative, asymptotic population estimates (MM Means) in EstimateS (Colwell, 2016). We collected precise information about spatial locations of individual frogs by recording all encounters on a highly detailed background map (Ringler et al., 2016) using pocketpcs (mobilemapper 10, Ashtech/Spectra Precision, Westminster, CO, USA) and the mobile GIS software arcpad 10 (ESRI, Redlands, CA, USA). Digital photographs of individually distinct ventral patterns were taken for later identification with the pattern matching software “Wild‐ID” (Bolger, Vance, Morrison, & Farid, 2011). Sex was determined by the presence (male) or absence (female) of vocal sacs.

In two sampling events (22 January–5 February 2013 and 19–26 March 2013), we collected and genetically sampled all the tadpoles (*N* = 734) inside the remaining ten artificial pools, as well as from four additional natural deposition sites that were discovered during our sampling (hereafter “fp” for “forest pool”). We also recorded the number of predatory dragonfly larvae that were present at each artificial pool site. While we did not detect any dragonfly predators in the forest pools, we scored predator presence inside all forest pools as missing data in case there were dragonflies present but undetected during observations of these natural pools.

Tissue was sampled from all tadpoles by clipping a small piece of tail and immediately preserving it in 96% ethanol (Ringler et al., 2014). Afterwards, all tadpoles were released back to their original pool. As tadpoles require between 40 and 50 days of aquatic development until metamorphosis (Weygoldt, 1980), the sampling interval of approximately 2 months ensured that all tadpoles recorded in the first sampling event had already emerged before the second session, thus preventing repeated sampling of single individuals. We verified this assumption via genetic similarity matching between samples (cf. Ringler et al., 2014).

### 2.4 Genotyping and parentage analyses

The genotypes of all adult individuals were already available from (Ringler et al., 2014), where all adult individuals were assigned to their respective founder tadpole from 2012 using genetic similarity matching. The tadpole release data from 2012 allowed us to reconstruct the natal pools of all adult individuals found in 2013. All tadpole samples collected for this study in 2013—representing the offspring of the founder adults of the island population—were genotyped at the same 14 highly variable microsatellite loci. Ambiguous loci were genotyped up to three times. For detailed PCR and genotyping protocols and characteristics of the microsatellite loci, see References: Ringler, Pašukonis, Hödl, and Ringler (2013a), Ringler et al. (2014), Ursprung et al. (2011).

Parentage assignment of adults and tadpoles from 2013 was carried out with colony2 (Jones & Wang, 2010); see Ursprung et al. (2011) for further details. The full likelihood model was used with medium precision allowing for polygamous mating in both sexes. All sampled adult males (*N* = 36) and females (*N* = 31) were treated as potential fathers and mothers, respectively. Only “Best (ML) Configuration” assignments with the maximum likelihood obtained at the end of the computation were used for subsequent analyses.

All inferred full siblings from the same sampling event were treated as belonging to the same clutch. Only in one case was a full‐sib batch considerably larger (*N* = 40) than the typical initial clutch size of ~20 eggs in *A. femoralis* (Ringler et al., 2012), and thus, we considered these tadpoles to stem from two separate clutches. We excluded all cases where only a single tadpole was assigned to a given parent pair to reduce the impact of potential false assignments; that is, we only considered clutches if they were represented by at least two full siblings in our analysis. We also excluded all tadpoles with simulated paternal genotypes provided by the COLONY analysis, as we could only determine movement distances for known individuals; tadpoles with simulated mothers were retained. We treated tadpoles from the same clutch that ended up in the same pool as one drop‐off event, representing a single deposition decision, assuming that male *A. femoralis* always shuttle multiple tadpoles at once (cf. Ringler et al., 2013b). As males were previously shown to also disperse their clutches (even within a single clutch) across multiple water bodies (Erich et al., 2015), we included clutch identity in our analyses to account for temporal and spatial autocorrelation of these data points. In previous studies, it was further shown that occasionally female *A. femoralis* will perform the tadpole transport in those cases where the respective fathers go missing during the period of clutch development (Ringler et al., 2013b; Ringler, Pašukonis, et al., 2015; Ringler, Hödl, et al., 2015). Our sampling design does not allow us to discern whether a male or a female deposited a certain tadpole. However, given that the frequency of females transporting tadpoles in the field is rather low (below 8%), that females mainly choose their mating partners in close distance to their resting sites (Ringler et al., 2012) and that most of the inferred fathers (71%) were still present after the second sampling, we assume that none of our inferences were significantly influenced by eventual cases of compensatory female tadpole transport.

After genotyping, some tadpole samples (*N* = 73) were excluded due to insufficient amplification success (i.e., more than four missing loci). Of 661 tadpoles that assigned parentage, 18 tadpoles were assigned to six “simulated” male genotypes and were thus excluded from further analyses. Tadpoles that were not assigned to another sibling (*N* = 31) were excluded from further analyses, ultimately leaving 612 tadpoles (of the original 734 tadpoles that were sampled) from 69 clutches (mean ± *SD* = 8.87 ± 7.70 tadpoles per clutch) for all remaining analyses.

### 2.5 Spatial analysis

**Figure 2:**
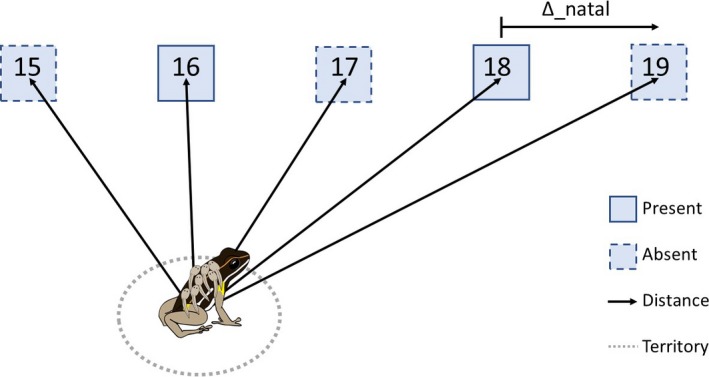
Schematic overview on the spatial parameters assessed for each tadpole sample. Squares with solid outlines represent available (“present”) pools and those with dashed outlines were removed (“absent”) pools. Distances were calculated from each male's territory centre to all pools. “Δ_natal” represents the distance in metres of a given drop‐off location to the male's natal pool (here pool #19) [Colour figure can be viewed at http://wileyonlinelibrary.com]

We determined the centroid point of all encounter locations of a given male, excluding all records during tadpole transport. If males changed territory location between the first and second sampling event, we calculated two separate centroids for the time periods before the first and second sampling events, respectively. We then calculated the straight‐line distance of the centroid points to all pool sites (“distance”). We also created the parameter “Δ_natal,” which was calculated as the distance in metres between a given drop‐off pool and the fathers’ natal pool. For example, a male whose natal pool was pool 19 (which had been removed) and that deposited tadpoles in pool 18 (Figure 2) would receive a score of Δ_natal = 10 for this deposition event, as pools were originally placed 10 m apart.

We investigated the distribution of tadpoles and dragonfly larvae across pools using the spatial coordinates of each single pool and the respective tadpole and dragonfly larvae counts in each of the sampling events. We performed a Moran's *I* test, using the package “ape” (Paradis, Claude, & Strimmer, 2004) in r (R Core Team, 2017).

To identify the parameters that best predict patterns of tadpole deposition across the installed artificial pools, we structured the data in a way that included all possible deposition options (i.e., all available 10 artificial pools) of a given tadpole transport event (i.e., the deposition of tadpoles from one single clutch across single or multiple pools) and noted whether the assigned father deposited tadpoles at a given pool or not (“dropoff”: yes = 1/no = 0). We further included the spatial variables “distance,” “Δ_natal” and the number of predatory dragonfly larvae present inside a given pool (see data set “dropoff”), as well as the male's identity (“maleID”), clutch identity (“clutchID”), whether a male had his natal pool still available or not (“natal_pool”: present/absent), the sampling event (first/second) and the geographic coordinates of the male's centroid point (“LongM,” “LatM”) in the data set. Using “dropoff” as the predictor, we fitted a spatial generalized linear mixed model (GLMM) using the function “corrHLfit” in the package “spamm” (Rousset & Ferdy, 2014) in r (R Core Team, 2017). As fixed effects, “distance,” “Δ _natal” and number of dragonfly larvae (“dragonflies”) were used. The parameters “clutchID” nested within “maleID” and “natal_pool,” as well as “sampling_event,” were used as random factors. In addition, to account for spatial autocorrelation between the location of the males’ territory and the fixed effects, a random effect with Matérn correlation function was added using the coordinates of the males’ centroid points. We further used the arguments “control.corrHLfit=list(optimizer=‘bobyqa’)” and “HLmethod= ‘PQL/L’” for fitting the model. A likelihood ratio test based on the ML fits of the full and the null model using the “fixedLRT” function was conducted to obtain *p*‐value estimates for the overall fit of the model, and confidence intervals were calculated for each of the fixed factors using the “confint” function of the spamm package.

### 2.6 Conditional inference trees and random forests

To investigate general deposition patterns across all frogs and samplings, we first fitted a Conditional Inference Tree (“ctree”) using the package “party” (Hothorn, Hornik, & Zeileis, 2006) in r (R Core Team, 2017). We used “dropoff” as the response variable and “dragonflies,” “distance” and “Δ_natal” as the partitioning variables considered for growing the ctree, as these had been identified as significant predictors of the response variable “dropoff” by the spatial GLMM. Although the ctree function does not allow for the specification of random factors, we fitted a tree by further adding the random factors “maleID,” “clutchID,” “natal_pool,” “sampling_event” and the coordinates of the males’ locations as fixed effects to allow splitting at those parameters. However, only “maleID” appeared at the lowest node of one branch, and the results of the analysis including random factors were highly consistent with analyses without random factors, suggesting that none of the random factors greatly influenced the hierarchical structure of parameters in our model.

We then iterated our classification trees by conducting a random forest analysis using the function “cforest” also available in the “party” package (Strobl, Boulesteix, Kneib, Augustin, & Zeileis, 2008) to evaluate the stability and reliability of the results from the full ctree analysis (all predictors and responses used). We used the same predictor variables as in the ctree analysis and ran 50 trees for each random forest. We then compared variable importance plots as provided by the model. A variable's importance was determined by the decrease in the predictive accuracy of the model when that variable is permutated. Tree‐based approaches have previously been proven highly useful for complex data sets, as they are robust to nonlinearity, non‐normality, multicollinearity and multiple interactions among explanatory variables (Quinn & Keough, 2002; Zuur, Ieno, Walker, Saveliev, & Smith, 2009). For much information on conditional inference trees and random forests, see text in Supporting Information.

### 2.7 Temporal analysis

**Figure 3:**
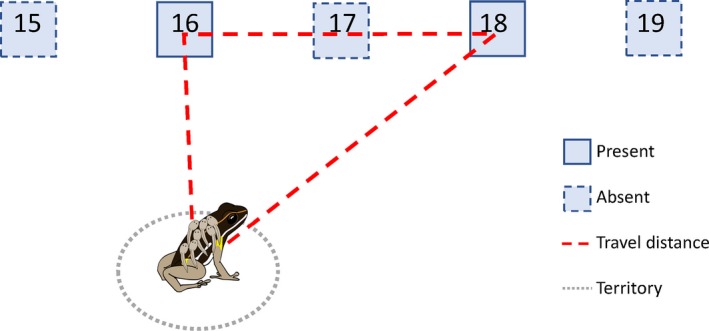
Schematic overview of one tadpole transport trajectory. This figure shows an example of one transport event, where a male deposited the tadpoles from one clutch in two pools (pools #16 and #18). The red dashed line represents the minimum travel distance the male could have taken [Colour figure can be viewed at http://wileyonlinelibrary.com]

We also investigated eventual changes in tadpole transport trajectories over time in both groups of males (natal pool present and absent). For this, we considered only actual deposition events (any record classified as “Yes” for the drop‐off category above) and restructured the data so that clutches constituted the unit of analysis (see data set “clutches”). For each clutch, we noted the minimum travel distance possible to visit all those pool sites (“travel_distance,” Figure 3), the average number of dragonfly larvae inside those pools (“Mean_dragonflies_”), the number of pools a male used to deposit the respective tadpoles (“N_pools_”) and the average spatial distance from a male's natal pool (“Mean_Δ_natal_”). This approach followed the rationale that *A. femoralis* generally pick up and transport entire clutches at once. Thus, if males deposit tadpoles from one clutch into several pools (i.e., different drop‐offs per clutch), they are likely to perform this in one single transportation event. As our sampling design could not identify the temporal sequence of single drop‐offs within one clutch transport, we used the minimum travel distance as a proxy for distribution effort.

We used “travel_distance,” “Mean_Δ_natal_” or “Mean_dragonflies_” as the response variables to fit linear mixed‐effect models (LMMs). The males’ identity was included as random effect in the model to account for nonindependence of data when single males were assigned to more than one clutch. In addition to random intercepts, random slopes were fitted for males to allow for intermale differences in behaviour (Barr, Levy, Scheepers, & Tily, 2013). As fixed effects, we used the categorical variables “sampling_event” (first or second) and “natal_pool” (present or absent), the latter coding whether a father's natal pool was still available or had been removed. We assured that assumptions of the models were not violated using diagnostic plots and statistics of the residuals.

To investigate eventual changes in the number of pools used to deposit single clutches across sampling events, we performed paired *t* tests by comparing the average number of pools individual males used to distribute their clutches in the first and second sampling events. This approach was taken (instead of a GLMM) as we had several males that were only observed in one of the sampling events, which impeded the calculation of reliable estimates when trying to fit a GLMM with a Poisson distribution. A Mann–Whitney *U* test was then used to compare the average number of pools between males that had their natal pool available and those that did not.

To test whether males whose natal pools had been removed were more exploratory and thus more likely to use forest pools than males whose natal pool was still present, we compared percentages of forest pool use across both male groups (data set “forestpools”), using a Mann–Whitney *U* test.

Normality of data was tested prior all analyses with Shapiro–Wilk tests, and nonparametric tests (e.g., Mann–Whitney *U* tests, Wilcoxon signed‐rank tests, Spearman's correlations) were applied when data significantly deviated from a normal distribution. Paired designs were used to test for differences within males across sampling events. All analyses were performed in the R Studio environment (RStudio Team, 2015).

## 3 RESULTS

In 2013, we sampled 36 male (estimated sampling coverage: 90%) and 31 female (estimated sampling coverage 67%) *A. femoralis* on the river island, representing the survivors of the initially released 1,800 tadpoles that had reached adulthood (Ringler et al., 2014). By comparing their microsatellite genotypes, we were able to match all adults to a corresponding tadpole sample. Consequently, we could identify the natal pool of all adult individuals present in 2013, although for this study only the information about the natal pools of males was relevant. Twenty of the males had their natal pool still available in 2013, while the natal pools of 16 males had been removed (see also Table S1 and Figure S1).

We sampled a total of 734 tadpoles from the 10 artificial pools (*N* = 664) and from four natural forest pools (*N* = 70). Pairwise relatedness values of all tadpole pairs were well below *r* = .8 (cf. Ringler et al., 2014), thus all representing unique genotypes. The total number of tadpoles sampled was similar between the first and second sampling events (*N*_first_ = 325, *N*_second_ = 409, Table S2). However, the number of tadpoles was not evenly distributed across space (first sampling: Moran's *I* = 0.15, p = 0.008, second sampling: Moran's *I* = 0.11, p = 0.017), as pools in the northern part of the island received significantly more tadpoles than pools located in the southern part (Figure S2). Twenty‐seven (75%) of the 36 males were assigned to a clutch via the parentage analysis. On average, there were eight tadpoles per clutch (mean ± *SD* = 8.87 ± 7.7). We recorded 124 drop‐offs that were deposited by 27 different males across artificial pools. Males used one to eight (mean ± *SD* = 3.22 ± 1.85) different pools (artificial and forest pools) to distribute their clutches. Males did not move significantly closer to or further away from their natal pool between first and second sampling events (Wilcoxon signed‐rank test: *N* = 35, *Z* = −1.048, *p* = .295).

### 3.1 Predator effect

We counted up to eight predatory dragonfly larvae inside a single pool (mean ± *SD* = 3.3 ± 2.49, range = 0–8 per pool, Table S2). The average number of dragonfly larvae present across pools was not significantly different between the first and second sampling events (Student's *t* test, *N* = 20, *t* = −0.350, *df* = 18, *p* = .730) and also did not change significantly within single pools over the course of the study (correlation: N_1_ = N_2_ = 10, Pearson's *r* = .783, *t*_8_ = 3.558, *p* = .007). However, the number of dragonfly larvae was not evenly distributed across space (first sampling: Moran's *I* = 0.16, *p* = .007, second sampling: Moran's *I* = 0.04, *p* = .087), as pools in the northern part of the island contained in general more dragonfly larvae than pools located in the southern part. The number of tadpoles per pool significantly decreased with increasing number of dragonfly larvae (correlation: *N* = 20, Spearman's rho = −0.578, *p* = .008, Figure S3), with no tadpoles present in pools with more than six dragonfly larvae.

### 3.2 Generalized linear mixed model

The likelihood ratio test revealed that the full model explained variations in the likelihood to deposit tadpoles (predictor “dropoff”) significantly better than the null model that included only random effects (χ^2^ = 87.217, *df* = 3, *p* < .0001). The spatial GLMM identified all three fixed factors as significantly affecting the probability of drop‐offs inside a given pool (Table S3). The drop‐off likelihood significantly increased with decreasing number of predatory dragonfly larvae (EM ± *SE* = −0.320 ± 0.050, *t* = −6.390, CI_95%_ = [−0.320; −0.214]), decreasing distance from a male's location to a given pool (EM ± *SE *= −0.018 ± 0.003, *t* = −6.080, CI_95%_ = [−0.017563; −0.017561]) and decreasing distance from a male's natal pool site (EM ± SE = −0.006 ± 0.002, *t* = −2.587, CI_95%_ = [−0.006; −0.001]).

### 3.3 Decision tree and forest modelling

**Figure 4:**
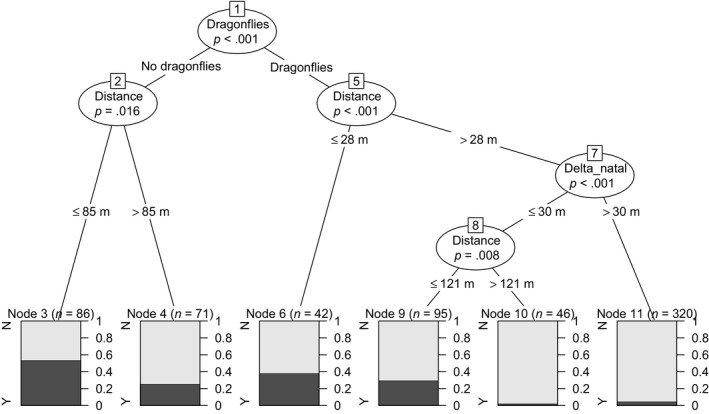
Conditional inference tree examining drop‐off choices made by male poison frogs. Pools that did receive a drop‐off (Y) versus those that did not (N) were best classified according to six categories. The highest drop‐off frequency (53.5%) was observed for pools that contained no dragonfly larvae and were in close spatial proximity to a male's territory. “dragonflies” = number of dragonfly larvae inside a given pool, “distance” = distance from the centre of a male's territory to a given pool, “delta_natal” = proximity to the male's natal pool. The labels in the figure were edited for clarity. The unedited version of this figure can be found in the supplementary materials

Conditional inference tree analysis revealed that the number of dragonflies (*p* < .001), followed by the distance between the pool and the male's current location (*p* < .001), and the distance from the male's natal pool site (“Δ_natal,” *p* < .001) were significant predictors for drop‐off frequency. Pools that did not contain any dragonfly larvae and that were located in spatial proximity to a male's territory (closer than 28 m) were particularly likely to receive tadpoles (left branch of the tree in Figure 4, 53.5% of pools that fell into this category were used for deposition). On the other end of the spectrum, pools that contained many dragonflies (more than six) and that were also far from both a male's current location (more than 85 m) as well as from the male's natal pool (more than 30 m) were least likely to receive tadpoles (right branch of the tree in Figure 4, only 4.7% of pools in this category received a drop‐off). The hierarchical structure of parameters is illustrated in Figure 4.

The random forest model confirmed the relative importance ranking of predictor variables as suggested by the spatial GLMM and the ctree in terms of their ability to predict whether tadpole drop‐off occurred. The number of dragonflies inside a pool had the highest predictive value (0.03), followed by spatial distance of a pool from a male's territory (0.019), and proximity to a male's natal pool (0.01). While our error rate for either classification averaged 17.27%, unsuitable pools that did not receive any drop‐offs were almost always correctly classified (error rate = 4%), but only one‐third of the pools that received drop‐offs were predicted to be drop‐off pools (error rate = 75%) in our model. This suggests that we can, with high confidence, predict pools that will not be used for tadpole deposition, but that a more complex suites of characteristics determine a suitable pool for deposition in this species.

### 3.4 Experience effect

**Figure 5:**
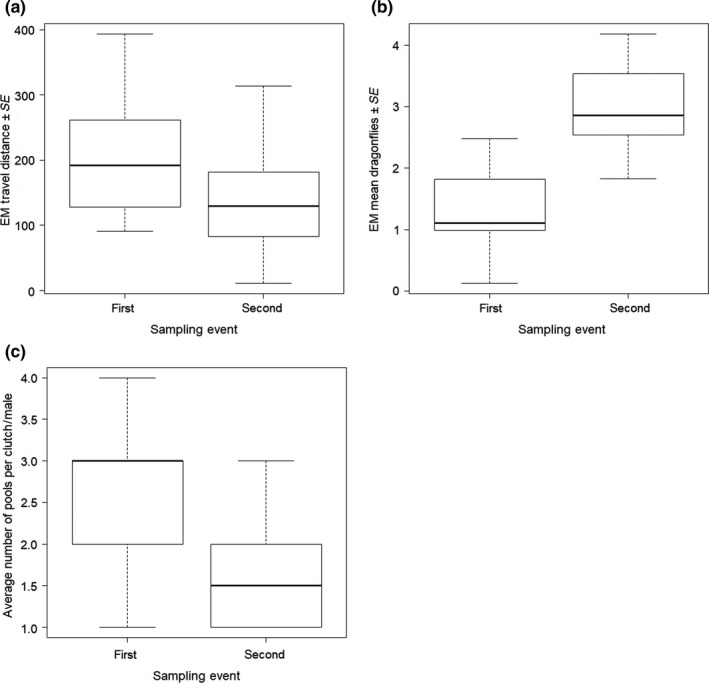
Temporal Effects of tadpole deposition. Boxplots showing (a) the estimated mean (EM) of the minimum travel distance of males, (b) the estimated mean number of dragonfly larvae in drop off pools and (c) the average number of pools males used to deposit clutches in the first and second sampling events

Linear mixed models (LMMs) showed that “travel_distance” was significantly influenced only by “sampling_event,” with significantly lower values in the second sampling event (EM ± *SE* = −79.84 ± 18.01, *df* = 48.40, *t* = −4.434, *p* < 0.0001, Figure 5a, Table S4). In turn, the mean number of dragonfly larvae inside drop‐off pools (“Mean_dragonflies_”) significantly increased from the first to the second sampling event (EM ± *SE* = 1.71 ± 0.46, *df* = 59.23, *t* = 3.68, *p* = .0005, Figure 5b, Table S4), indicating that males were more tolerant towards pools containing dragonfly larvae later in the season. The presence or absence of a male's natal pool did not have a significant effect on either of the two response variables (travel_distance: EM ± *SE* = −38.62 ± 36.83, *df* = 24.32, *t* = −1.05, *p* = .305; “Mean_dragonflies_”: EM ± *SE* = 0.66 ± 0.56, *df* = 21.56, *t* = 1.19, *p* = .246). The location of drop‐off pools relative to a male's natal pool “Mean_Δnatal_” did not significantly differ between the two samplings (EM ± *SE* = −6.99 ± 4.73, *df* = 42.16, *t* = −1.48, *p* = .147) nor did it differ between both male groups (natal pool present versus absent; EM ± *SE* = 0.58 ± 21.48, *df* = 24.57, *t* = 0.03, *p* = .98).

The number of pools used by males to distribute tadpoles of a single clutch significantly decreased from the first to the second sampling event (paired *t* test: N_1_ = N_2_ = 13, *t*_12_ = 4.46, *p* = .0008, Figure 5c). However, the number of pools did not significantly differ between males that had their natal pool available when compared to those that did not (Mann–Whitney *U* test: *N*_present/absent_ = 14/11, *U* = 74, *p* = .889).

### 3.5 Use of forest pools

Sixteen drop‐offs from ten different males, with one to four drop‐offs per male, occurred in forest pools. Contrary to our prediction, we found that males whose natal pools had been removed were not more likely to use forest pools than males whose natal pool was still present (Fisher's exact test: four of 15 males versus six of 12 males, *p* = .2566). We also did not find any significant difference in the percentage of forest pools used across both male groups (Mann–Whitney *U* test: *N* = 27, *U* = 110, *p* = .2716). Forest pools in which males deposited tadpoles were not significantly closer to the respective males’ territories than the artificial pools (forest pools: mean distance ± *SD* = 71.38 ± 59.77 m, closest artificial pools: mean distance ± *SD* = 33.98 ± 23.58 m; paired *t* test: N_1_ = N_2_ = 10, *t *= 2.035, *p* = .072); forest pools were the closest available water bodies in only three of 16 drop‐off events (Figure S5).

## 4 DISCUSSION

In the present study, we used an introduced poison frog population confined to a river island during its colonization phase to investigate strategies for the exploitation of reproductive resources. The combination of introducing tadpoles to a previously uninhabited river island and their genetic tracking until adulthood allowed us to identify natal pools of all adult survivors in the subsequent breeding season. The use of novel methods and statistical tools also shed light on the interactions of biotic and abiotic factors and the hierarchical decision patterns utilized by this species during tadpole transport. We found male *A. femoralis* exhibiting a hierarchical, albeit flexible and context‐dependent decision process when choosing water bodies for tadpole deposition, supporting the hypothesis that unpredictable environments favour the evolution of dynamic resource‐use strategies.

### 4.1 Predation threat vs. spatial proximity

Drop‐off likelihood significantly decreased in the presence of predators, suggesting that adult males do actively avoid pools that contain dragonfly larvae, corroborating the findings of Erich et al. (2015), that also identified a negative relationship between dragonfly larvae and number of tadpoles inside pools. Theoretically, such a negative relationship could also result from a high predation rate on deposited tadpoles rather than from males choosing not to deposit within pools that contain dragonfly larvae. However, we consider this not to be a major factor in our study for several reasons. First, many anuran species are able to detect aquatic predators and adjust their deposition behaviour accordingly to avoid those pools for breeding (Binckley & Resetarits, 2003; Touchon & Worley, 2015), which also has been found in *A. femoralis* (McKeon & Summers, 2013). Second, all of our analyses were performed on the drop‐off or clutch level, and thereby controlled for variable drop‐off and clutch sizes. A drop‐off would only have gone undetected if dragonfly larvae had consumed all tadpoles of this drop‐off. We consider this possibility very unlikely, given the high number of tadpoles per clutch and the high number of represented clutches per pool. Our total sampling of tadpoles inside pools was a further safeguard against missing any drop‐off. Consequently, we are confident that the significant negative relationship between dragonfly larvae and tadpoles is indeed a result of the direct avoidance of tadpole predators in pools by adult frogs.

Pools that contained dragonfly larvae were particularly unlikely to be chosen for deposition (Figure 4), and even less so if they were further away (>85 m) from a males’ territory. However, males did tolerate dragonfly larvae if pools were particularly close (<28 m) to their territory. This highlights the trade‐off between current reproductive success, as larval survival is affected by pool quality, and future reproductive success. Tadpole transport is expensive in terms of time and energy. As males need to leave their territory in order to reach a suitable water body, they risk losing their territory to other male competitors or mating opportunities with females in the meantime. Moreover, increased visibility during movement might increase predation risk and thus considerably decrease their future reproductive success. In male *A. femoralis*, lower survival chances will not only reduce their potential to invest in future matings, but also their ability to care for remaining clutches inside their territory. Consequently, males are expected to keep transportation time and effort to a minimum, and/or time transport to coincide with periods of low predator and conspecific activity (Beck et al., 2017; Ringler et al., 2013b). However, if pool quality (e.g., in terms of predation threat) falls below a certain threshold, benefits of avoiding that pool might counterbalance elevated costs of extended transporting efforts.

Both the spatial GLMM and random forest analyses confirmed that the number of predatory dragonfly larvae was the most important factor predicting where drop‐offs occurred. The classification matrix as provided by this analysis further showed that our variables could consistently predict which pools were highly unlikely to receive drop‐offs, for example pools with many dragonfly larvae that were far from both male territories and natal sites. However, the model was relatively weak in predicting which pools could receive drop‐offs, which probably reflects the variation in individual preferences across males, their differential tolerance thresholds for distance and number of predators and their tendency to disperse tadpoles across multiple pools.

The evolution of specific predator detection and avoidance abilities is particularly likely for species with offspring that are highly vulnerable to a specific predator, when the predator occurs patchily throughout the landscape and is relatively common and stationary (i.e., predictable) and when other predator‐free resources are available (Binckley & Resetarits, 2002; Blaustein, Kiflawi, Eitam, Mangel, & Cohen, 2004). Some anurans are capable of perceiving danger from tadpole predators even when they have no prior experience with that particular species (Downie, Livingstone, & Cormack, 2001), indicating that avoidance of predator cues may be innate. In our study, the presence and abundance of dragonfly larvae in single pools did not significantly change over time, but there was high variation across pools. We expect large reproductive benefits in males that are attentive to predator presence in pools. Dragonfly larvae are known to be top predators of amphibian eggs and larvae, and to either fully or partially prey on tadpoles, the latter causing severe limb malformations (Relyea & Hoverman, 2003). Our results show that presence of dragonfly larvae was one of the most important factors for assessing pool quality by *A. femoralis*, with high dragonfly larvae counts (above 6) ruling out tadpole presence.

### 4.2 Natal philopatry and temporal effects

Our findings show that male *A. femoralis* exhibited a strong preference for returning to their natal area, even if closer pools were available and despite some dragonfly larvae inside those natal pools. However, we also found that a few weeks later clutches were distributed with a concurrent decrease in the number of used pools and of the overall distances travelled. Thus, later in the season males chose fewer pools and/or pools that were closer to their current location (i.e., their territory), despite an elevated presence of predators in those pools. These findings indicate that tadpole transport trajectories are optimized over time in terms of time and energy expenditure, probably through a highly efficient use of spatial learning and memory. Contrary to our prediction, we did not find any differences in tadpole distribution between males that had their natal pool still available and males that did not. We hypothesize that males may already have information about various water bodies in their local area early in the season and that they immediately adapt their tadpole transport trajectories accordingly. This idea is supported by the fact that there were no detectable differences in forest pool use or in the average number of pools used between both groups of males. Males that had their natal pool removed did not exhibit more pronounced exploration patterns, probably because they were already aware of sufficient alternative water bodies.

Time and energy efforts associated with the exploration of novel resources might be particularly costly in complex and fluctuating habitats such as tropical rainforests. Individuals therefore might benefit from returning to known sites such as their natal pool. This might, on the one hand, serve as a “safe” backup in situations where resources are highly limited. On the other hand, strong (natal) philopatry may also result in maladaptive behaviour if individuals repeatedly return to low‐quality habitats (Matthews & Preisler, 2010). Previous studies have provided strong evidence that *A. femoralis* males have a mental representation of their local area and use spatial memory to access reproductive resources (Pašukonis et al., 2014, 2016; Ringler et al., 2013b). However, another recent study showed that movement trajectories in tadpole‐transporting frogs were always highly goal‐directed and did not show any signs of exploration during either tadpole transport or homing (Beck et al., 2017). These findings also suggest that male *A. femoralis* do not use an integrative comparison strategy where multiple pools are visited first and the best site is only selected afterwards (i.e., Bayes comparison). Instead, they employ an immediate strategy where pools are visited sequentially and judged whether they meet some minimum criteria (e.g., the threshold approach; Bell, 1991). When and how exactly frogs learn about specific pools within their local habitat remains open for further investigation. The recent discovery that tadpole transport can be experimentally initiated via adding tadpoles to a frogs’ back could serve as a powerful technique to answer these questions (Pašukonis et al., 2017).

### 4.3 Larval competition

In many anuran species, larval density is found to negatively impact larval fitness due to increased competition for limited resources such as food and space, which may increase time to, and decrease size at metamorphosis, ultimately reduce larval survival (Relyea & Hoverman, 2003; van Allen, Briggs, McCoy, & Vonesh, 2010). Cannibalism on tadpoles or eggs is also common in many dendrobatid tadpoles (Caldwell & de Araújo, 1998; Schulte & Mayer, 2017; Summers & Symula, 2001), resulting in strong avoidance of already occupied water bodies in these species (Schulte & Lötters, 2014). As *A. femoralis* tadpoles do not prey on each other (Weygoldt, 1980, obs. in captivity; also E. Ringler, personal observation) and nutrients and space also do not seem to constitute limiting factors in most breeding pools used by this species, male *A. femoralis* do not avoid placing their offspring in pools that already contain conspecific tadpoles. The most attractive pool (#14 in the second sampling event) contained 152 tadpoles in total, representing 23 drop‐offs by 14 different males. On the one hand, high numbers of tadpoles inside pools were linked to low numbers of dragonfly larvae. Thus, males could potentially use tadpole density as a proxy for predator presence. On the other hand, high tadpole density might further serve to reduce the risk of predation on individual tadpoles via a dilution effect on predators (Buxton & Sperry, 2016; Buxton, Ward, Sperry, & Foster, 2017). Consequently, we suggest that high numbers of tadpoles inside pools might be a reliable signal for pool stability and quality in *A. femoralis*.

## 5 CONCLUSIONS

In complex natural environments, resources used by animals differ in predictability, availability and quality. Multiple biotic and abiotic factors influence breeding site selection behaviour in the field (see Buxton & Sperry, 2016; Buxton et al., 2017 for amphibians). Our results show that the trade‐off between current and future reproductive success can shape resource‐use strategies during parental care. The underlying decision‐making most likely occurs sequentially at different hierarchical levels. Our findings suggest that preferences and strategies remain dynamic throughout the course of the breeding season to optimize associated trade‐offs. Investigating these strategies and trade‐offs will provide a deeper understanding of mental capabilities and decision‐making strategies in rainforest frogs.

## CONFLICT OF INTEREST

The authors declare no competing interests.

## AUTHOR CONTRIBUTION

E.R. and M.R. designed the study. E.R., R.M. and M.R. collected the data. E.R. and P.B.‐B. performed the molecular analysis. E.R., M.R., G.S. and R.J.H. analysed the data. E.R. drafted the manuscript. All authors contributed to the writing of the final version of the manuscript.

## DATA ACCESSIBILITY

Supporting information and data are available from the Dryad Digital Repository: https://doi.org/10.5061/dryad.5st48g8.
