# Supplementary material for: Hierarchical decision‐making balances current and future reproductive success
Source: Mol Ecol. 2018 Apr 23;27(9):2289–301. doi: 10.1111/mec.14583 (PMC5969290; doi:10.1111/mec.14583)
Supplement: Supplementary file 1 [file MEC-27-2289-s001.pdf]

Table S1. List of all adult males present on the island in 2013. ‘Natalpool’ indicates which of the 20 initial pools each male originated from, and ‘Pool_present’ indicates whether the respective pool is still present after fall 2012, or not (Y = yes; N = no).

Male	Natalpool	Pool_present
**13-m-001**	12	Y
**13-m-002**	6	Y
**13-m-003**	17	N
**13-m-004**	14	Y
**13-m-005**	12	Y
**13-m-006**	19	N
**13-m-007**	9	N
**13-m-008**	2	Y
**13-m-009**	16	Y
**13-m-010**	2	Y
**13-m-011**	7	N
**13-m-012**	4	Y
**13-m-013**	10	Y
**13-m-014**	2	Y
**13-m-015**	7	N
**13-m-016**	8	Y
**13-m-017**	18	Y
**13-m-018**	18	Y
**13-m-019**	11	N
**13-m-020**	9	N
**13-m-021**	17	N
**13-m-022**	10	Y
**13-m-023**	17	N
**13-m-024**	5	N
**13-m-025**	1	N
**13-m-026**	2	Y
**13-m-027**	17	N
**13-m-028**	18	Y
**13-m-029**	2	Y
**13-m-030**	16	Y
**13-m-031**	7	N
**13-m-032**	17	N
**13-m-033**	2	Y
**13-m-034**	7	N
**13-m-035**	19	N
**13-m-036**	12	Y

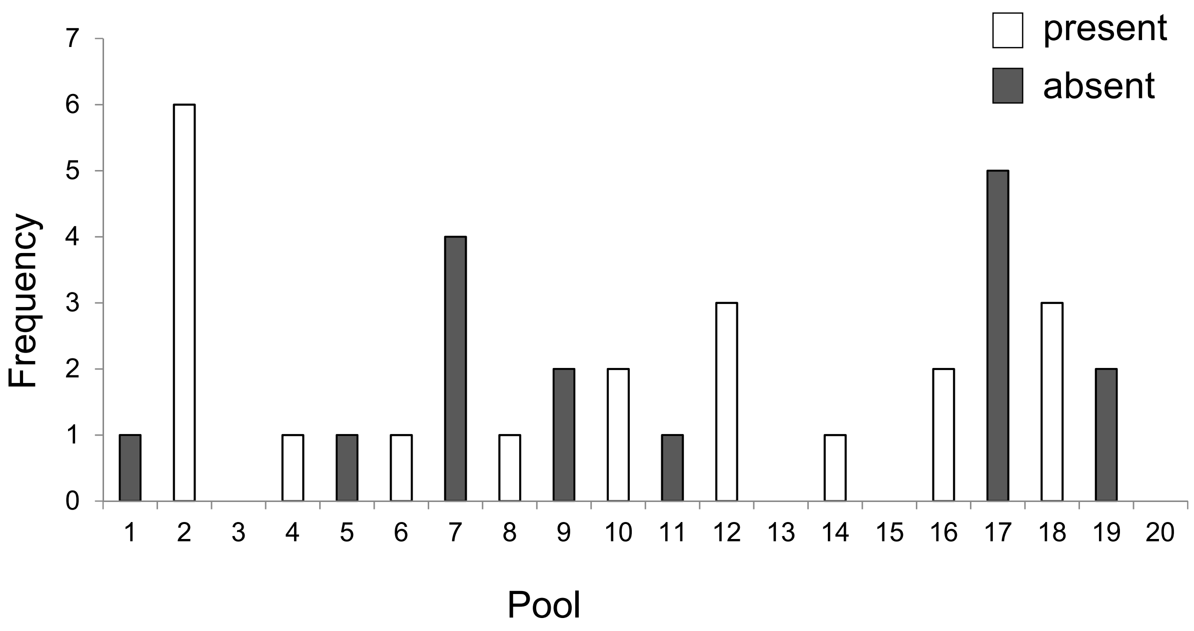

Figure S1. Number of male survivors across the 20 initially installed pools. After removing half of the pools (odd numbers), only the 10 pools with even numbers remained, resulting in 20 males that had their natal pool still available, and 16 males that had their natal pool removed.

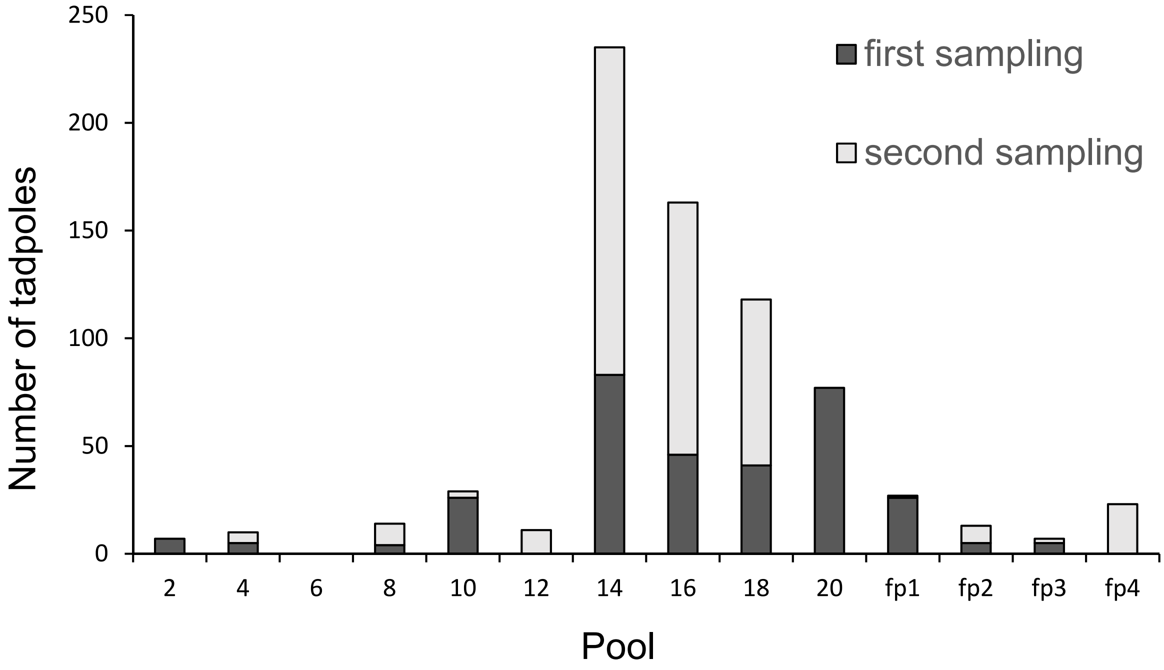

Figure S2. Tadpole counts across water bodies. Number of tadpoles sampled across the 10 remaining pools in 2013, as well as from the natural forest pools. Dark shading refers to tadpoles sampled in the first, and light shading refers to tadpoles sampled in the second sampling event.

Table S2. Number of dragonfly larvae and tadpoles recorded per pool and sampling event.

	First sampling		Second sampling	
**Pool**	**# dragonfly larvae**	**# tadpoles**	**# dragonfly larvae**	**# tadpoles**
**02**	3	7	5	0
**04**	4	5	3	5
**06**	8	0	7	0
**08**	5	4	3	10
**10**	2	26	4	3
**12**	6	0	5	11
**14**	3	83	6	152
**16**	0	46	0	117
**18**	0	41	0	77
**20**	0	77	2	0
**Forest pools**	NA	36	NA	34

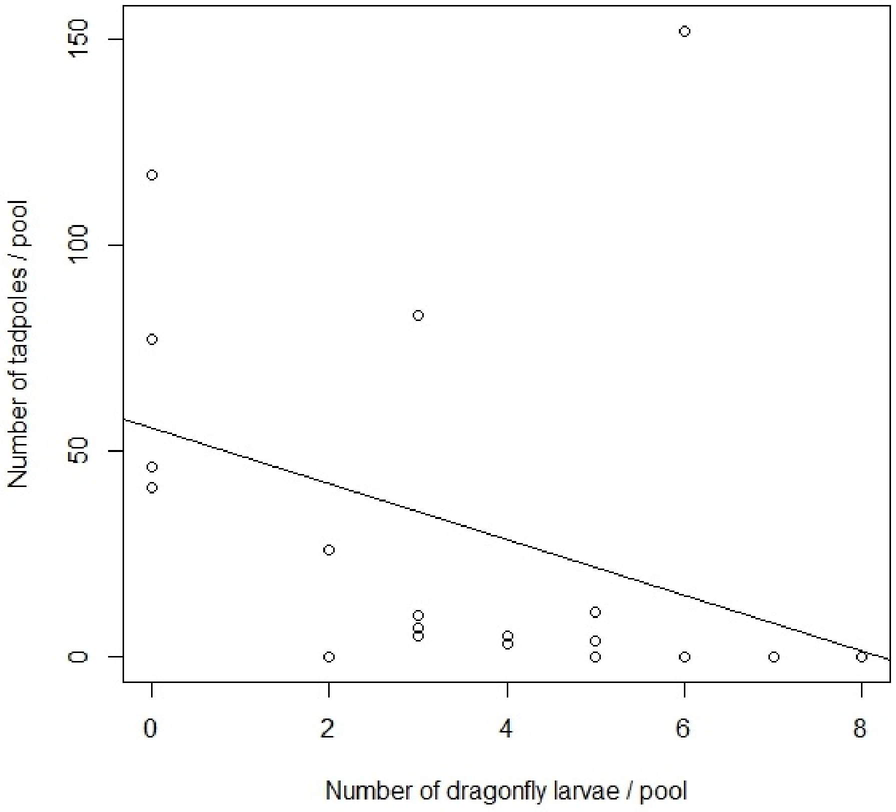

Figure S3. Predator effect. The number of tadpoles observed was significantly negatively related with the number of dragonfly larvae deposited inside a given pool.

Table S3. Results from the spatial GLMM.

Dropoff	EM	SE	*t* value	95% CI
(Intercept)	1.456	0.467	3.114	
Dragonflies	-0.320	0.050	-6.390	[-0.320,-0.214]
Distance	-0.018	0.003	-6.080	[-0.017563,-0.017561]
Δ_natal	-0.006	0.002	-2.587	[-0.0059;-0.0005]

The likelihood ratio test showed that the full model explained significantly more variation than the null model ($Chi^2 = 87.217$, df = 3, $p < 0.0001$).

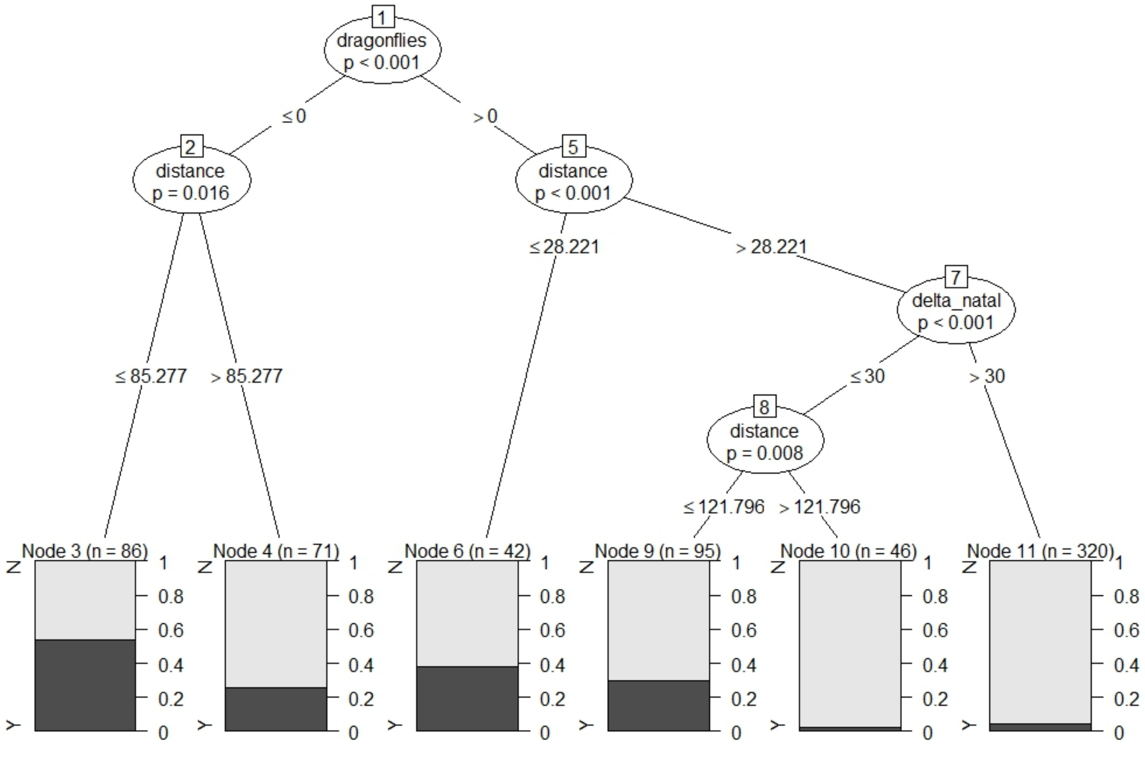

Figure S4. Unedited Conditional Tree. Pools that did receive a drop-off (Y) versus those that did not (N) were best classified according to six categories. The highest drop-off frequency (53.5 %) was observed for pools that contained no dragonfly larvae and were in close spatial proximity to a male's territory. 'dragonflies' = number of dragonfly larvae inside a given pool, 'distance' = distance from the centre of a male's territory to a given pool, 'delta_natal' = deviation from the male's natal pool.

Table S4. Results from the LMMs.

**Mean travel distance**	**EM**	**SE**	**df**	***t* value**	***p***
(Intercept)	223.59	27.66	36.14	8.08	<0.0001
Sampling event (1st vs. 2nd)	-79.84	18.01	48.40	-4.43	<0.0001
Natal pool (present vs. absent)	-38.62	36.83	24.32	-1.05	0.305
**Mean number of dragonflies**	**EM**	**SE**	**df**	***t* value**	***p***
(Intercept)	0.92	0.49	42.36	1.89	0.065
Sampling event (1st vs. 2nd)	1.71	0.46	59.23	3.68	0.0005
Natal pool (present vs. absent)	0.66	0.56	21.56	1.19	0.246
**Mean Δ natal**	**EM**	**SE**	**df**	***t* value**	***p***
(Intercept)	70.47	14.66	27.47	4.81	<0.0001
Sampling event (1st vs. 2nd)	-6.99	4.73	42.16	-1.48	0.147
Natal pool (present vs. absent)	0.58	21.48	24.57	0.03	0.979

Sampling event: 1st event was set as reference; natal pool: 'present' was set as reference.

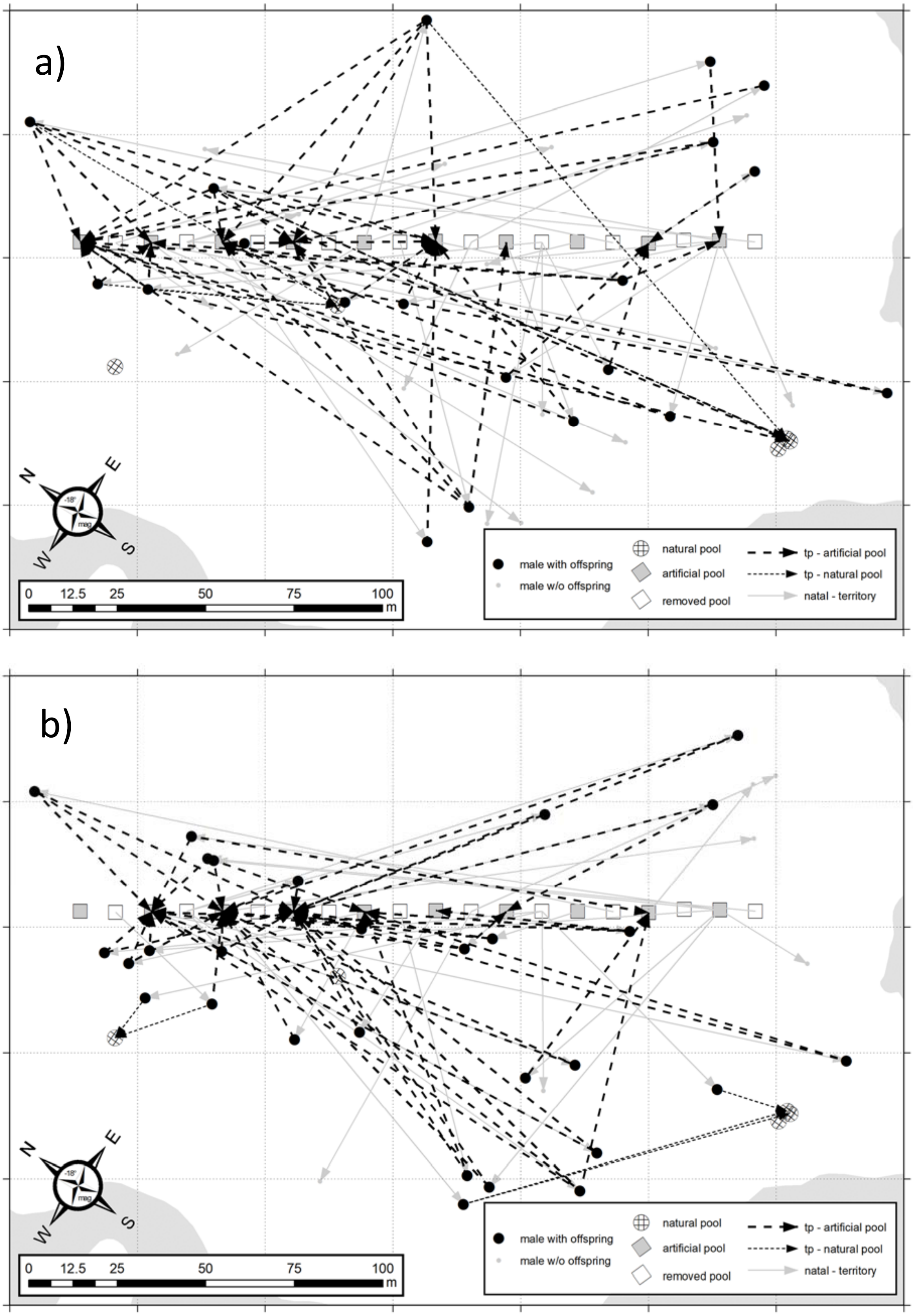

Figure S5. Tadpole distribution across water pools in the (a) first and (b) second sampling event. Arrows with large dashed lines indicate tadpole drop-offs at artificial pools, arrows with small dashed lines indicate drop-offs at forest pools, and light grey arrows show migration patterns from a male's natal pool site to its territory location.

Conditional Tree and Random Forest Modeling

Decision tree modeling is a powerful tool for both exploratory data analysis and predictive modeling (Breiman, 1984, 2001). Originating from machine learning and artificial intelligence research (Michalski, Carbonell, & Mitchell, 2013), it has also proven useful in ecological (Guisan & Zimmermann, 2000) and medical research (Bennett & Hauser, 2013), yet the potential of tree and random forest modeling in behavioural ecology and animal cognition research has so far received less consideration.

Tree-based models use recursive partitioning to split the data into sub-groups via a set of predictor variables. The overall objective is to find a combination of decision rules that provide an informative and robust hierarchical classification model. Each split is assessed for each potential explanatory variable, resulting in a maximum between-variation, and a minimum within-variation of groups (Clark & Pregibon, 1992) (It should be noted, however, that splits in explanatory variables at precise values may represent only approximations of real thresholds). While single trees hierarchically structure the data according to a set of predictor variables, random forests assess the classification error rate and estimate the relative importance of each predictor in the model (Liaw & Wiener, 2002). Therefore, they use a randomized iterative algorithm in which multiple trees are constructed using randomly selected subsets of the data. The error rate of the model is based on the combined error rate of all iterations. Variables are ranked in order of importance in classifying the data, which corresponds to the decrease of classification accuracy when a variable is randomly permuted (Breiman, 2001). Random forest approaches make no *a priori* assumptions about the relationship between the response and predictor variables, are less susceptible to spatial autocorrelation, and perform especially well with large datasets (Breiman, 2001; Strobl, Malley, & Tutz, 2009).

The advantages of this approach over additive or general linear modeling are that (i) there is no requirement for linearity and normality in explanatory variables, (ii) multicollinearity of explanatory variables does not affect the model (iii) interactions among terms can be easily identified and visualised; (iv) the number of explanatory variables that can be included is unlimited; and (v) it is relatively robust to outliers and noise (Quinn & Keough, 2002; Zuur, Ieno, & Smith, 2007). However, tree-branched models have the drawback that splitting is biased in favour of explanatory variables in which more splitting is possible, and they can easily be over-fitted that may require subjective 'pruning' methods (Quinn & Keough, 2002; Strobl, Boulesteix, Kneib, Augustin, & Zeileis, 2008; Zuur et al., 2007). In contrast, Conditional Inference Trees and Forests (Hothorn, Hornik, Strobl, & Zeileis, 2010; Hothorn, Hornik, & Zeileis, 2006), which also use binary recursive partitioning, but use a conditional inference framework, are not affected by over-fitting and also less susceptible to differences in the scale of measurement of predictor variables (Strobl, Boulesteix, Zeileis, & Hothorn, 2007).

REFERENCES

Bennett, C. C., & Hauser, K. (2013). Artificial intelligence framework for simulating clinical decision-making: s Markov decision process approach. *Artificial Intelligence in Medicine*, *57*, 9–19. doi:10.1016/j.artmed.2012.12.003

Breiman, L. (1984). Classification and regression trees: Wadsworth International Group.

Breiman, L. (2001). Statistical Modeling: The Two Cultures (with comments and a rejoinder by the author). *Statistical Science*, *16*, 199–231. doi:10.1214/ss/1009213726

Clark, L. A., & Pregibon, D. (1992). Statistical models in S. *chapter Tree-Based Models*, 377–419.

Guisan, A., & Zimmermann, N. E. (2000). Predictive habitat distribution models in ecology. *Ecological Modelling*, *135*, 147–186. doi:10.1016/S0304-3800(00)00354-9

Hothorn, T., Hornik, K., Strobl, C., & Zeileis, A. (2010). Party: *A laboratory for recursive partytioning.*

Hothorn, T., Hornik, K., & Zeileis, A. (2006). Unbiased recursive partitioning: a conditional inference framework. *Journal of Computational and Graphical Statistics*, *15*, 651–674. doi:10.1198/106186006X133933

Liaw, A., & Wiener, M. (2002). Classification and regression by randomForest. *R news*, *2*, 18–22.

Michalski, R. S., Carbonell, J. G., & Mitchell, T. M. (2013). Machine Learning: *An Artificial Intelligence Approach*: Springer Berlin Heidelberg. Retrieved from https://books.google.at/books?id=-eqpCAAAQBAJ

Quinn, G. P., & Keough, M. J. (2002). Experimental design and data analysis for biologists: Cambridge University Press.

Strobl, C., Boulesteix, A.-L., Kneib, T., Augustin, T., & Zeileis, A. (2008). Conditional variable importance for random forests. *BMC bioinformatics*, *9*, 307. doi:10.1186/1471-2105-9-307

Strobl, C., Boulesteix, A.-L., Zeileis, A., & Hothorn, T. (2007). Bias in random forest variable importance measures: Illustrations, sources and a solution. *BMC bioinformatics*, *8*, 25. Retrieved from https://doi.org/10.1186/1471-2105-8-25

Strobl, C., Malley, J., & Tutz, G. (2009). An introduction to recursive partitioning: rationale, application, and characteristics of classification and regression trees, bagging, and random forests. *Psychological Methods*, *14*, 323–348. doi:10.1037/a0016973

Zuur, A. F., Ieno, E. N., & Smith, G. M. (2007). Analysing ecological data. *Statistics for Biology and Health*. New York, NY,USA: Springer Science + Business Media, LLC.
